# Supplementary material for: The impact of scabies in tent cities in Kahramanmaraş after the Turkish earthquakes: oral pharmacologic treatment efficacy
Source: PeerJ. 2024 Oct 14;12:e18242. doi: 10.7717/peerj.18242 (PMC11485099; doi:10.7717/peerj.18242)
Supplement: Supplemental Information 3 [file peerj-12-18242-s003.docx]

# The Prevalence of Scabies in Tent Cities in Kahramanmaraş After the Turkish Earthquakes: How Effective is Oral Pharmacological Therapy?

**1) Gender:** a)Female b)Male

**2) Age:**........

**3) Education:**

a) Illiterate b) Primary education c) Secondary education-High school d) University or postgraduate

**4) Accommodation area:**

a) Container b) Tent (single) c) Tent City d) Other...

**5) Number of people living in the household**:............

**6) Do you have a previously diagnosed skin disease?**

a)yes b)no

**-** **If yes, are you receiving treatment?**

a)yes b)no

**7) What symptoms are you currently experiencing with your disease?**

a) Pruritis b) Rash c) Subcutaneous crawling sensation d) Fever

**8) How many days you have had symptoms?**.......

**9) How many people in the household have symptoms?**.......

**10) Severity of symptoms before ivermectin treatment (0 none, 10 very severe)**

a) Pruritis…. b) Rash…. c) Subcutaneous crawling sensation…. d) Fever….

**11)** **Is there a lesion? If so, where on the body?**

a. Fingers b. Wrists c. Hands d. Forearm e. Umbilicus f. Back

g. Foot h. Leg i. Face j. Neck k. Scalp l. Other.....

**12)** **Ivermectin (scabies tablets) treatment start date?** ...............

**13)** **Severity of symptoms on day 5 after first dose of ivermectin treatment? (0 none, 10 very severe)**

a) Pruritis…. b) Rash…. c) Subcutaneous crawling sensation…. d) Fever….

**14) Is there recovery of lesions after the first dose of ivermectin treatment?**

a) no recovery b) partial recovery c) complete recovery

**15) Date of second dose?** ...............

**16) Severity of symptoms on day 5 after second dose of ivermectin treatment? (0 none, 10 very severe)**

a) Pruritis…. b) Rash…. c) Subcutaneous crawling sensation…. d) Fever….

**17) Is there recovery of lesions after the second dose of ivermectin treatment?**

a) no recovery b) partial recovery c) complete recovery

**18) Lesion type or types?**

a)Papules b)Vesicles c) Nodules d)Eczematization e)Excoriations
