# Supplementary material for: The impact of scabies in tent cities in Kahramanmaraş after the Turkish earthquakes: oral pharmacologic treatment efficacy
Source: PeerJ. 2024 Oct 14;12:e18242. doi: 10.7717/peerj.18242 (PMC11485099; doi:10.7717/peerj.18242)
Supplement: Supplemental Information 4 [file peerj-12-18242-s004.docx]

**Türkiye'de Yaşanan Depremler Sonrası Kahramanmaraş'taki Çadır Kentlerde Uyuz Hastalığının Yaygınlığı: Oral Farmakolojik Tedavi Ne Kadar Etkili?**

**1) Cinsiyet:** a)Kadın b)Erkek

**2) Yaş:**........

**3) Eğitim durumu:**

a) Okuryazar değil b) İlköğretim c) Ortaöğretim-Lise d) Üniversite veya lisansüstü

**4) Yaşam alanı:**

a) Konteyner b) Çadır (tek kişilik) c) Çadırkent d) Diğer...

**5) Hanede yaşayan kişi sayısı:**............

6**) Daha önce teşhis edilmiş bir cilt hastalığınız var mı?**

a)evet b)hayır

**- Cevabınız evet ise, tedavi görüyor musunuz?**

a)evet. b)hayır

**7) Şu anda hastalığınızla ilgili hangi belirtileri yaşıyorsunuz?**

a) Kaşıntı b) Kızarıklık c) Cilt altı yürüme hissi d) Ateş

**8) Kaç gündür semptomlarınız var?** .......

**9) Hanede kaç kişide semptom var?** .......

**10) İvermektin tedavisinden önce semptomların şiddeti (0 hiç yok, 10 çok şiddetli)**

a) Kaşıntı…. b) Kızarıklık…. c) Cilt altı yürüme hissi…. d) Ateş….

**11) Bir lezyon var mı? Varsa, vücudun neresinde?**

a. Parmaklar b. El bilekleri c. Eller d. Ön kol e. Göbek f. Sırt

g. Ayak h. Bacak i. Yüz j. Boyun k. Saçlı deri l. Diğer.....

**12) Ivermectin (uyuz tableti) tedavisine başlama tarihi?** ...............

**13) İlk doz ivermektin tedavisinden sonraki 5. günde semptomların şiddeti? (0 hiç yok, 10 çok şiddetli)**

a) Kaşıntı…. b) Kızarıklık…. c) Cilt altı yürüme hissi…. d) Ateş….

**14) İlk doz ivermektin tedavisinden sonra lezyonlarda iyileşme var mı?**

a) iyileşme yok b) kısmi iyileşme var c) tam iyileşme

**15) İkinci dozun tarihi?** ...............

**16) İkinci doz ivermektin tedavisinden sonraki 5. günde semptomların şiddeti? (0 hiç yok, 10 çok şiddetli)**

a) Kaşıntı…. b) Kızarıklık…. c) Cilt altı yürüme hissi…. d) Ateş….

**17) İkinci doz ivermektin tedavisinden sonra lezyonlarda iyileşme var mı?**

a) iyileşme yok b) kısmi iyileşme var c) tam iyileşme

**18) Lezyon tipi veya tipleri?**

a)Papül b)Vezikül c)Nodül d)Ekzematizasyon e)Ekskoriasyon
